# Marine phosphate availability and the chemical origins of life on Earth

**DOI:** 10.1038/s41467-022-32815-x

**Published:** 2022-09-02

**Authors:** Matthew P. Brady, Rosalie Tostevin, Nicholas J. Tosca

**Affiliations:** 1 Department of Earth Sciences, University of Cambridge, Cambridge, CB2 3EQ UK; 2 Department of Geological Sciences, University of Cape Town, Cape Town, 7700 South Africa

**Keywords:** Element cycles, Origin of life, Environmental chemistry, Geochemistry

## Abstract

Prebiotic systems chemistry suggests that high phosphate concentrations were necessary to synthesise molecular building blocks and sustain primitive cellular systems. However, current understanding of mineral solubility predicts negligible phosphate concentrations for most natural waters, yet the role of Fe^2+^, ubiquitous on early Earth, is poorly quantified. Here we determine the solubility of Fe(II)-phosphate in synthetic seawater as a function of pH and ionic strength, integrate these observations into a thermodynamic model that predicts phosphate concentrations across a range of aquatic conditions, and validate these predictions against modern anoxic sediment pore waters. Experiments and models show that Fe^2+^ significantly increases the solubility of all phosphate minerals in anoxic systems, suggesting that Hadean and Archean seawater featured phosphate concentrations ~10^3^–10^4^ times higher than currently estimated. This suggests that seawater readily met the phosphorus requirements of emergent cellular systems and early microbial life, perhaps fueling primary production during the advent of oxygenic photosynthesis.

## Abstract (web summary)

Phosphate is critical for all life on Earth but its origins have remained enigmatic. Experiments indicate that phosphate may have been abundant in ancient Fe-rich seawater, providing a crucial ingredient for the origins of life on Earth.

## Introduction

The universal and deep-seated importance of phosphorus in biology, from its role as a structural component to the basic energy currency of all living cells, has long suggested that it was incorporated early in the history of life^1^. However, beyond its biological utility, recent advances in prebiotic systems chemistry have shown that at high concentration (~0.1–1 mol/kg), phosphate performs an array of chemical functions; it facilitates the selective formation of amino acids, lipid precursors, and nucleotides from one multicomponent reaction network^2–4^, assuming a central role in the chemical origins of life.

Nevertheless, soluble phosphate is widely thought to have been scarce on the prebiotic Earth. Apatite-group minerals, the dominant repository of phosphate within Earth’s crust, are insoluble under most conditions^5^, and the presence of Fe^2+^, solubilised under ancient O_2_-poor atmospheres, is thought to have limited phosphate concentrations to ~10^−7^ mol/kg in most natural waters and seawater^5,6^. These difficulties have motivated proposals for alternative, or more reactive, sources of phosphorus^7–9^, and for non-aqueous solvents that could have facilitated phosphorylation^10,11^, but plausible sources for these components have not been identified.

As a consequence, scenarios for the chemical origins of life on Earth are currently predicated on specific environments where phosphate may have reached concentrations suitable for prebiotic synthesis. For example, aqueous alteration of schreibersite ((Fe,Ni)_3_P), an accessory mineral in meteorites, yields highly soluble reduced P species^3,5,12^ but not organophosphate molecules^5^, though the products can be oxidised to phosphate through UV photolysis in the presence of hydrogen sulfide^13^. Dissolved phosphate may also have been concentrated in acidic volcanic springs^14^ or in alkaline lakes^15^; however, the possibility of prebiotic synthesis in these environments does not address the problem of how phosphate continued to sustain primitive cellular systems and early microbial life^16^.

Despite its importance, the availability of dissolved phosphate on the early Earth is poorly constrained. Phosphate solubility in multicomponent solutions (i.e., seawater) is dependent on ionic strength and cation composition because Ca^2+^ and Mg^2+^ associate strongly with phosphate anions^17^, but data for Fe^2+^ are unavailable. For example, aqueous Fe(II)-phosphate complexation has not been characterised above acid pH, and although the precipitation of Fe(II)-phosphate minerals may have been favoured on the early Earth^3,5,6,18,19^, solubility has not been quantified above pH ~4.7–5.6 or as a function of ionic strength and cation composition^20^. The lack of a data-calibrated geochemical framework has led to variable predictions of phosphate solubility in modern anoxic systems that commonly differ by several orders of magnitude^21^.

Here, we determine the solubility of Fe(II)-phosphate in synthetic seawater as a function of pH and ionic strength, integrate these observations into a thermodynamic model that predicts phosphate concentrations across a range of aquatic conditions, and validate these predictions against modern anoxic sediment pore waters. Experiments and models indicate that Hadean and Archean seawater featured phosphate concentrations ~10^3^−10^4^ times higher than currently estimated, which in turn re-shapes current understanding of the environmental conditions that may have been compatible with prebiotic synthesis.

## Results

### Experimental constraints on Fe(II)-phosphate solubility

**Fig. 1:**
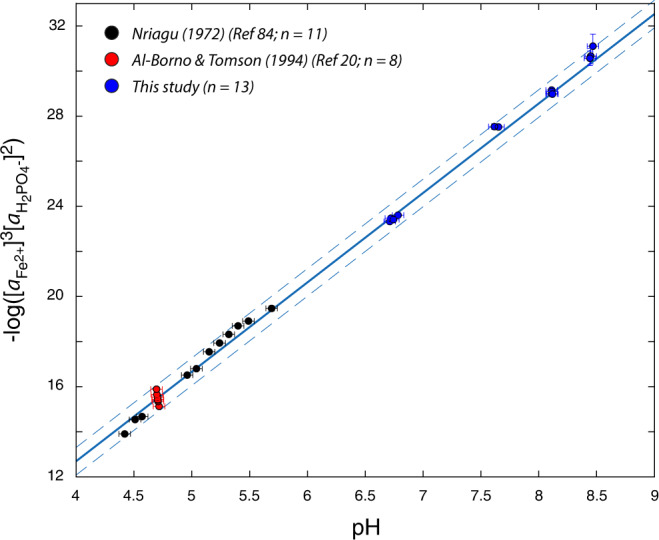
Solubility of vivianite at 25 ^o^C. Optimisation of new and existing solubility data in the aqueous iron phosphate system yields a value of 35.79 +/− 0.09 for the solubility product of vivianite (pK_sp_; solid line; dashed lines indicate 95% confidence intervals of entire dataset) for vivianite, as well as equilibrium constants for aqueous Fe-phosphate species (Supplementary Tables 1 and 2). This experimentally calibrated model represents, within reported error, all available experimental observations conducted at varying pH, ionic strength and media composition. Error bars indicate an analytical error (two standard deviations from the mean; refs. 20, 84).

To quantify the behaviour of dissolved phosphate in prebiotic environments, we determined the solubility of Fe(II)-phosphate (vivianite) in synthetic seawater solutions at 25 ^o^C in the absence of O_2_ and as a function of pH and ionic strength (“Methods”). In contrast with common assumptions^5,6^, these solubility data, when combined with previous determinations at 25 ^o^C, show that the apparent solubility of vivianite increases several orders of magnitude between pH 4 and 8.5 (Fig. 1). This in turn indicates that strong complexation between aqueous Fe^2+^ and phosphate anions controls solubility as pH increases and phosphoric acid species de-protonate.

### Optimising and validating thermodynamic models of Fe(II)-phosphate solubility

**Fig. 2:**
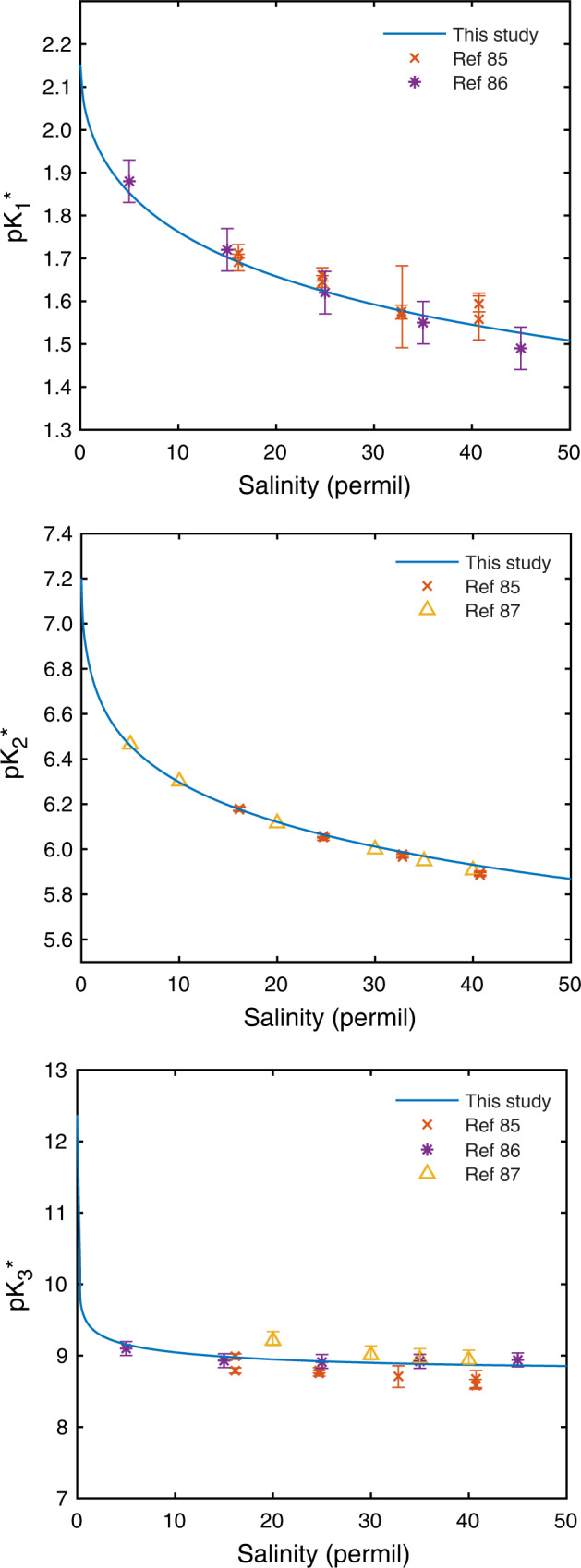
Stoichiometric dissociation constants of phosphoric acid in seawater media as a function of salinity. At 0 permil salinity, calculated stoichiometric dissociation constants are equivalent to the infinite dilution values listed in Supplementary Table 2. Error bars indicate an analytical error (two standard deviations from the mean; refs. 85–87).

In order to accurately predict dissolved phosphate concentrations across a range of conditions, we integrated these experimental data into a Pitzer ion interaction-based thermodynamic model for aqueous phosphate in the presence of several major ionic components, including Fe (“Methods”). Experimental data were used to retrieve equilibrium constants that describe aqueous Fe(II)-phosphate complexation across pH and ionic strength in order to achieve a consistent representation of phosphate solubility in anoxic systems (“Methods” and Supplementary Table 1). The model reproduces, within analytical error, measured stoichiometric dissociation constants of phosphoric acid in seawater media as a function of ionic strength (Fig. 2), and shows that Ca-, Mg- and, most importantly, the presence of Fe^2+^, increases the solubility of all phosphate minerals in anoxic systems. Experiments and calculations also reveal that vivianite solubility decreases with decreasing salinity, establishing a chemical mechanism to explain the reported locus of vivianite deposition in modern brackish and lacustrine systems, and its rarity in normal marine and hypersaline environments^22^.

To assess the accuracy of our model in predicting dissolved phosphate concentrations in multicomponent systems, we calculated the saturation state of Ca- and Fe(II)-phosphate minerals for natural waters that co-exist with vivianite (“Methods”). These results show, for example, that when vivianite is present, pore water saturation states are poised precisely at equilibrium vivianite solubility, in turn reconciling variable reports of phosphate mineral saturation in anoxic waters (Supplementary Figs. 1–3).

## Discussion

In order to test the hypothesis that Ca- and/or Fe(II)-phosphate mineral precipitation strongly limited phosphate concentrations on the prebiotic Earth, we used the model to examine phosphate concentrations in seawater as a function of pH, salinity, cation concentration and dissolved inorganic carbon (DIC). Because maximum phosphate concentrations in anoxic seawater are sensitive to pH and total Fe(II) concentration, [Fe^(II)^_Total_], constraints on seawater Fe(II) concentrations are required. Higher atmospheric *p*CO_2_ and the absence of skeletal biota imply that Precambrian seawater likely featured increased [DIC] and [SiO_2_(aq)], consistent with sedimentological and geochemical characteristics of ancient rocks^23,24^. It follows that maximum [Fe^(II)^_Total_] is likely to have been controlled by competition between Fe(II)-carbonate and/or -silicate minerals. Laboratory experiments on the nucleation and growth of Fe(II)-minerals from anoxic SiO_2_(*aq*)-rich seawater show that Fe(II)-silicate (greenalite) precipitation controls [Fe^(II)^_Total_] despite high DIC and strong supersaturation with respect to FeCO_3_ minerals^25^. Those data delineate the pH range over which various Fe(II)-minerals precipitate, in turn providing experimentally calibrated constraints on [Fe^(II)^_Total_] in SiO_2_(aq)- and CO_2_-rich seawater.

**Fig. 3:**
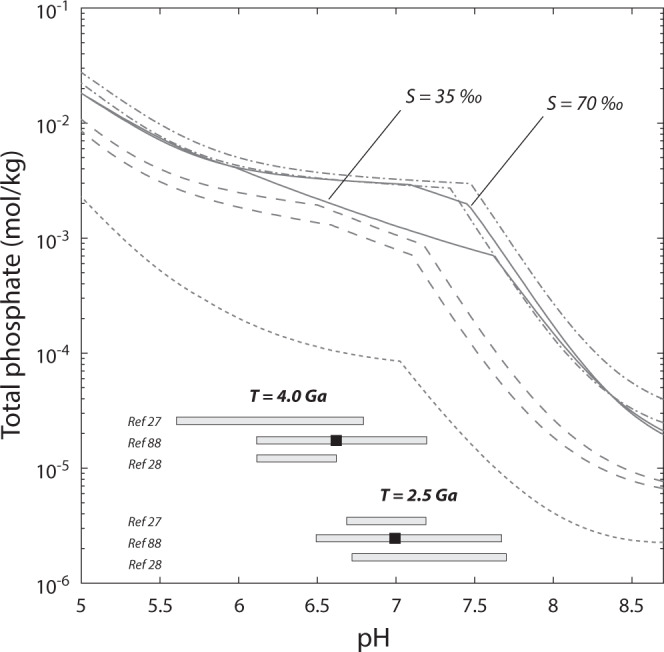
Phosphate solubility in anoxic seawater at 25 ^o^C. At pH >7.2–7.7, total aqueous phosphate is limited by greenalite and octacalcium phosphate (OCP) solubility, whereas at pH <7.2–7.7, it is limited by OCP and vivianite solubility. Solid lines adopt modern cation composition ([SO_4_] = 0) at varying salinities. Dashed lines encompass end-member cation compositions for prebiotic seawater (dashed dot lines: two high-Mg and high-Ca fluid compositions derived from the interaction between komatiite and CO_2_-rich fluids at two different water:rock ratios;^29^ long dashed lines: two high-Mg and high-Ca fluid compositions derived from the interaction between basalt and CO_2_-rich fluids at two different water:rock ratios;^29^ short dashed line: modelled high-Ca and low-Mg composition assuming elevated hydrothermal water flux and modern proportions of Mg removal at near- and off-axis vent fluids^28^). All calculations maintain equilibrium with 0.1 bar atmospheric pCO_2_^23,28^. Model-estimated pH values for Hadean and late Archean seawater are shown as grey bars (refs. 27, 28, 88).

Because Ca and Mg also complex strongly with phosphate anions, predicting phosphate solubility requires constraints on their relative concentrations in ancient anoxic seawater. In the absence of direct observational constraints, some studies have hypothesised that modern seawater cation ratios were established in the Hadean^26,27^, whereas others have attempted to constrain the cationic composition of seawater by examining the role of high-temperature interactions with the primitive crust^28,29^. These latter studies indicate that across a range of temperatures, relatively acidic CO_2_-rich fluids lead to higher carbonate and Mg-silicate solubilites^29,30^ and therefore are associated with higher [Mg] than modern systems, consistent with severely Mg-depleted silicified komatiites, which are thought to represent the products of low-temperature off-axis hydrothermal circulation^31,32^. Nevertheless, whether Hadean CO_2_-rich systems were a net Mg source or sink depends on the [Mg]/[Ca] of seawater and on the low and high-T hydrothermal effluent. Thus, we adopt high-Ca/low-Mg and high-Ca/high-Mg end-member compositions in order to bracket uncertainty in the cationic composition of prebiotic seawater (Fig. 3).

Together, experimental constraints on marine Fe concentrations indicate that maximum phosphate concentrations would have been limited by Ca-phosphate mineral solubility. Although carbonate fluorapatite (CFA) is the dominant Ca-phosphate mineral preserved in modern and ancient marine sediments, experimental and observational data indicate that its formation proceeds via nucleation of octacalcium phosphate (OCP), and subsequent recrystallisation to CFA^33,34^. Thus, depending on pH, OCP and/or Fe(II)-phosphate solubility together set maximum limits on marine phosphate concentrations. Overall, for Fe(II)-bearing waters at pH ~6.2–7.2^23,28^ total P ranges from ~200–4000 μmol/kg, three to four orders of magnitude higher than currently estimated^5,6,18^.

**Fig. 4:**
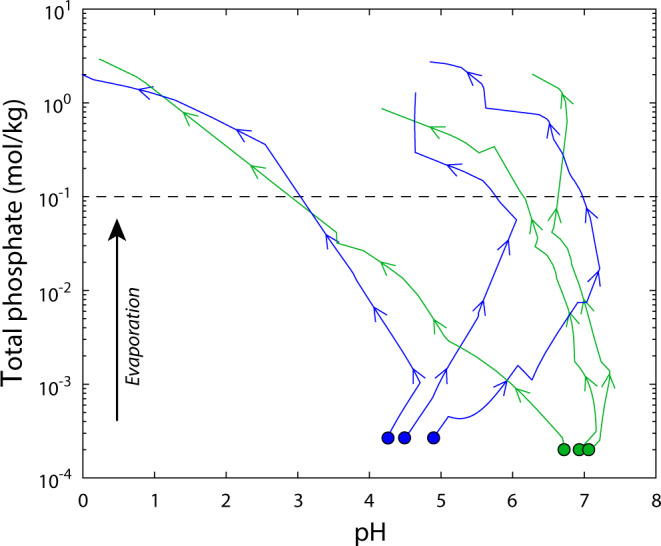
Evaporation of anoxic waters on early Earth and Mars. Reaction path calculations predict that evaporation of anoxic seawater (green dots) and dilute waters influenced by interaction with martian basalt (blue dots) produce phosphate-rich fluids at a range of pH values. The evolution of pH is in turn dictated by the relative proportion of total alkalinity (ALK) to the molar quantity of cations partitioned into carbonate minerals. Seawater calculations adopt modern cation composition ([SO_4_] = 0), [Fe_T_] = 200 μmol/kg, amorphous silica saturation and pCO_2_ = 0.1 bar^23,28^. Basaltic water composition is constrained by experiments conducted with synthetic martian basalt^82^ and maintains equilibrium with 1 bar CO_2_ (“Methods”). From left to right, the initial ALK/[Ca + Mg] for basaltic waters is 0.12, 1.33 and 2.20 and for seawater is 0.11, 0.74 and 0.96. Dashed line indicates the approximate minimum total aqueous phosphate concentration required for prebiotic synthesis experiments^2–4^.

Reaction path calculations show that evaporation of prebiotic seawater would have further concentrated phosphate, but we find that pH evolution of the evaporating fluid is strongly dictated by the relative proportion of total alkalinity (ALK) to the molar quantity of cations partitioned into carbonate minerals. This is because under likely atmospheric *p*CO_2_ and pH conditions of prebiotic seawater^23^, Ca- and Mg-carbonate minerals precipitate early in the evaporation sequence, and if insufficient residual alkalinity is present following carbonate precipitation, pH decreases as evaporation and carbonate precipitation proceeds (Fig. 4). For example, evaporation of relatively low alkalinity seawater increases phosphate concentrations by a further three orders of magnitude while pH decreases from ~6.5 to <3 (Fig. 4). Conversely, if sufficient ALK remains after carbonate precipitation, as suggested by sodium carbonate pseudomorphs in Archean rocks^35^, pH increases and/or remains high upon evaporation. Under these conditions, CaCO_3_, and amorphous silica form early in the evaporation sequence, followed by greenalite, Mg-carbonate and chloride salts. This pathway leads to a dominantly Mg–Cl rich fluid with >1 mol/kg total phosphate buffering the solution to pH 6–7 (Fig. 4).

Our results suggest that Hadean oceans could have delivered a crucial prebiotic feedstock molecule to a wide array of marine systems, where interaction with reactive solid and aqueous species may have facilitated key steps toward biosynthesis^36^. Beyond the marine realm, dry land is often invoked to concentrate feedstock chemicals and leverage selective photochemistry afforded by solar radiation^2–4,13^. However, geophysical evidence suggests that dry land may have been spatially limited on the early Earth, in turn limiting P sources to non-marine environments^37,38^. Our results suggest that ancient oceans could have constantly supplied phosphate and other marine components (i.e., via relative sea-level change, groundwater-seawater interactions and/or sea spray aerosol deposition) to terrestrial environments regardless of the magnitude of continental inputs. Subsequent evaporation of these fluids would have further concentrated aqueous phosphate, and although environments associated with evaporation may have assumed only local importance, repeated cycles of sea-level change may have promoted evaporation across diverse geochemical conditions depending on relatively minor fluctuations in alkalinity modulated by marine, continental, and/or atmospheric inputs (Fig. 4 and Supplementary Figs. 4–6). This chemical diversity of phosphate-rich fluids could have both expanded the combinatorial possibilities for prebiotic synthesis^4^ and helped to sustain primitive cellular systems. For example, the dilution and evaporation cycles that promote aqueous phosphate concentration also drive pH oscillations which facilitate RNA strand separation, peptide elongation, and fatty acid vesicle formation^16,39,40^. Our results also suggest that Earth’s ancient oceans would have readily met phosphorus requirements for self-replicating cellular systems. The estimated range for total marine phosphate concentrations exceeds the concentration required to drive several biochemical reactions (e.g., glycolysis) and approaches the concentration of intracellular phosphate in living cells, which is tightly linked to basic metabolic requirements^41^. This in turn may indicate that the mechanisms by which prokaryotes accumulate and transport inorganic phosphate from modern oceans, mediated largely by Mg-phosphate complexation^42^, arose from evolutionary adaptions to decreasing phosphate concentrations over Earth’s early history.

New observational data confirm the solubility relations predicted in Fig. 3 and suggest that phosphate-rich oceans may have persisted through much of the Archean eon until the great oxidation event (GOE). However, the upper limit on marine phosphate concentrations is subject to the magnitude of other sinks which may have been operative across this interval, specifically the production of Fe(III)-oxides and/or primary production. Conventional models for the early P cycle hypothesise that Fe(III)-oxides would have served as an important P sink^6^ given experimental evidence for UV photo-oxidation. However, the magnitude of Fe(III)-oxide production before the GOE has proven difficult to quantify from ancient iron-rich chemical sediments. High resolution microscopy^43,44^, geochronology^45^ and paleo-magnetic studies^46^ suggest that much of the Fe(III)-oxides in Archean iron formation are likely to derive from metamorphic oxidation. At the same time, nanoscale petrographic analysis indicates that the Fe(II)-silicate greenalite is likely to have formed as a pervasive early precipitate from ancient seawater, given its ubiquity in Fe-rich sedimentary rocks that span nearly the entire Archean stratigraphic range^36,43,44^. These observations are consistent with mass balance constraints on global oxidant budgets^47^, and with phylogenetic analyses suggesting a relatively late origin for oxygenic photosynthesis^48^. Although primary production may have buried more P per mole of C in an oxidant-limited biosphere^49^, the magnitude of this sink relative to P sources also remains uncertain. Theoretical considerations suggest that total C fluxes through the Archean mantle–crust–ocean–atmosphere may have been higher than today^23^, with rates of global primary production expected to have been much lower (100–1000×)^47^; these factors must be reconciled with the TOC record of Archean shales^19^. Together, considering experimental evidence for enhanced P input fluxes from anoxic seafloor alteration^50^, merging available constraints with our new experimental data suggests that phosphate-rich Archean oceans are likely to have featured marine Ca-phosphate (and perhaps Fe-phosphate) precipitation as a dominant output flux. This is consistent with the identification of ubiquitous apatite nanoparticles, deposited with greenalite, throughout Archean-aged (3.46–2.46 Ga) chemical sediments^51^, in turn constraining total aqueous phosphate to ~10^−4 ^mol/kg (Fig. 3), approximately three orders of magnitude higher than the modern ocean.

It is also widely assumed that Fe(II)-phosphate precipitation from anoxic oceans would have limited net primary productivity of microbial ecosystems and organic C burial, which may have attenuated rates of atmospheric O_2_ accumulation^6,18,19^. However, our experimentally calibrated model of phosphorus geochemistry, in combination with new observations of apatite-rich sediments deposited immediately before the GOE^51^, suggest the possibility that oxygenic photosynthesis emerged against a phosphate-rich backdrop, potentially triggering a cascade of biogeochemical responses.

Finally, our results inform the search for life on other planets, including Mars. Aqueous alteration of the early martian surface is thought to have supplied aquatic systems with abundant ferrous iron and phosphate^52^, and both data and models suggest that geochemical environments may have been commonly associated with high ALK/[Ca + Mg]^53^. Our calculations indicate that these characteristics would have generated phosphate-rich fluids with near-neutral pH upon evaporation, commonly producing vivianite, amorphous silica, and Ca- and Mg-carbonate minerals (Fig. 4 and Supplementary Figs. 7–9). The resulting assemblage, consistent with orbital data returned from Jezero Crater^54^, provides a specific signature for ancient environments where the synthesis of key biomolecules may have been feasible. Together, the chemistry of ancient Fe-rich environments ensured that phosphate was an integral part of prebiotic and early biotic chemical landscapes, endowing life on Earth with a crucially versatile building block.

## Methods

### Experimental and analytical methods

Experiments were carried out in a Coy polymer anaerobic chamber, purged with a 4% H_2_/96% N_2_ gas mixture. An anoxic atmosphere was maintained over the course of the experiments using a Pd catalyst (which reacted any trace O_2_ with H_2_ to form H_2_O) and CaSO_4_ desiccant connected to a fan box which provided continuous circulation. A Coy gas analyser constantly measured the composition of the atmosphere within the chamber, and it was maintained at <1 ppm O_2_(*g*) throughout the experiments. Deionised water was deoxygenated prior to reagent preparation by purging with O_2_-free N_2_ gas for >45 min^55^ before being transferred into the anaerobic chamber, where it was left stirring for a further 24 h to allow remaining oxygen to degas. The temperature was maintained at 25 +/− 1 ^o^C. Each experiment was carried out in 1 L low-density polyethylene (LDPE) bottles which were triple wrapped in Al foil to prevent photolysis reactions driven by ultraviolet light emitted from fluorescent lights^56^.

Stock solutions were prepared by dissolving metal-chloride powders (FeCl_2_·4H_2_O, MgCl_2_·6H_2_O and KCl; ≥99% Acros Organics, Sigma-Aldrich) in deoxygenated DI water. The Fe^2+^ stock solution was treated with Fe^0^ powder (≥99%; Sigma-Aldrich) and HCl to reach a pH <2 and left for 24 h. The goal of this treatment was to chemically reduce any Fe^3+^ that may have existed in stock FeCl_2_·4H_2_O powder prior to the transfer of the reagent to the glovebox. The Fe^0^ powder was then removed by running the stock solution through a custom-made polycarbonate polymer container with a high-strength N52 Nd magnet inserted in the wall. Soluble phosphate was introduced into the experiments by diluting sodium phosphate standard stock solutions (1 mmol/kg) into the working experiment solution.

Before the addition of FeCl_2_ to the experiment, which marks the start of the experiments, pH was set by the addition of 0.5 M deoxygenated NaOH. Experiments were run until apparent solubility equilibrium was achieved, which was defined by invariant (within analytical error) Fe^2+^ and phosphate concentration profiles typically sustained greater than a 24–48 h period. Once experiments were complete, solutions were vacuum filtered (0.1 μm) in the anaerobic chamber and rinsed in 250 ml deoxygenated deionised water. The filter membrane and associated solids were dried under the low-humidity, anoxic atmosphere within the chamber, where they were then transferred into sample vials which were wrapped in Al foil.

Major cation concentrations were determined by sampling over the course of the experiment. Each 0.2 mL sample was extracted in the glovebox and filtered with a 0.22 micron syringe filter and diluted (by weight) in HNO_3_; analysis of all solutions was performed via ICP-OES at the University of Cambridge. Total dissolved phosphate concentrations were measured spectrophotometrically using the HACH PhosVer method, which promotes the reaction of orthophosphate with molybdate in an acid medium to produce a phosphomolybdate complex. This complex is then reduced by ascorbic acid, which produces an intense molybdenum blue colour (quantified by the intensity of the 880-nm absorption).

Solid materials were analysed by powder X-ray diffraction (XRD) using a PANalytical Empyrean Series 2 X-ray diffractometer with a Co Kα source, operated at 40 kV and 40 mA, and scanned between 5 and 80 deg 2Θ; step size 0.026°. Powders were loaded on single crystal silicon sample holders and sealed with Kapton film within the glovebox to minimise oxidation during XRD analysis. Final X-ray diffractograms are shown with “background” patterns removed after subtracting several analyses performed on blank sample holders covered with Kapton film. Powder X-ray diffractograms were subject to Rietveld refinement in order to confirm phase identification and to constrain the incorporation of any other minor elements (i.e., Mg). Rietveld refinements were performed using GSAS-II^57^ and began with structural models for vivianite reported by ref. 58.

Fourier transform infrared spectroscopy (FT-IR) analyses were performed on powdered products and were acquired in transmission mode using a PerkinElmer Frontier mid-IR spectrometer. FT-IR transmission measurements were collected on optically transparent KBr pellets with a sample:KBr ratio of ∼1:200. KBr powder and solid materials were ground and compressed into pellets at 7 t and analysed within 15 min of exposure to air (Fe(III)-O vibrations were closely monitored to ensure minimal oxidation had taken place). Measurements were collected from 400 to 4000 cm^−1^ at 1 cm^−1^ resolution using a deuterated triglycine sulphate (DTGS) detector with a KBr window and beamsplitter. After analysis, pellets were left in an oven at 150 ◦C for 24 h to remove any adsorbed H_2_O that gave a broad FT-IR absorption peak between 3200 to 3700 cm^−1^ and analysed again.

### Development and optimisation of the thermodynamic model

For all calculations performed in this paper, we calculated ion activity coefficients using the Pitzer ion interaction model framework^59^. Within this framework, the interaction between a given cation and anion can be accounted for by parameterising cation–anion, cation-cation, and anion–anion interactions among all system components. However, for some strongly associating cation–anion pairs, it is possible to account for interactions by incorporating ion pairing within the Pitzer ion interaction framework^60^. Here we have accounted for interactions between major phosphate anions and Ca^2+^, Mg^2+^ and Fe^2+^ through the explicit incorporation of ion pairs, as described in more detail below.

The model utilises Pitzer ion interaction coefficients for the major sea salts (i.e., H, Na, K, Ca, Mg, Fe^2+^, Cl, SO_4T_, HCO_3_, CO_3_, OH) from the compilation of ref. 61. Activity coefficients for Fe^2+^ were calculated by accounting for Fe^2+^ interactions with Cl^-^ and SO_4_^2–^ using Pitzer parameters from refs. 62, 63. These are used by refs. 59, 61, 64, but the latter study did not use binary Pitzer coefficients for Fe^2+^-HSO_4_^-^, which we adopt here (from ref. 63). We account for strong interactions between Fe^2+^ and OH^−^ and carbonate species through ion pairing, using formation constants and Pitzer parameters from ref. 64. As described in that study, this approach closely estimates ion activity coefficients for various Fe^2+^ complexes that have been determined in ionic media of varying ionic strength.

To estimate the ion activity coefficients of phosphate species, we adopt recommended dissociation constants for phosphoric acid at infinite dilution from ref. 65. We utilise Pitzer parameters accounting for the interaction between phosphate species and Na^+^ and K^+^ from ref. 62, doublet interaction parameters for phosphate species and chlorine from ref. 66, and parameters for triplet interactions from refs. 66, 67. To account for strong interactions between phosphate species and Ca^2+^ and Mg^2+^, we include formation constants for six Ca- and Mg-phosphate species from refs. 68, 69.

Together, the model estimates, with reasonable accuracy, measured total ion activity coefficients in seawater at 25^ o^C and 35‰ salinity. In addition to closely representing measured total ion activity coefficients for the principal phosphoric acid species in seawater, the model also provides a good fit to available data for stoichiometric (or total) dissociation constants for phosphoric acid in seawater as a function of salinity (Fig. 2). Although we have taken a different approach than ref. 70 in estimating ion activity coefficients of phosphate species in seawater, our estimates generally agree with theirs but the inclusion of ion pairing provides a closer fit to stoichiometric dissociation constants measured in seawater media as a function of ionic strength (Fig. 2).

The chemical equilibrium model was used to optimise all newly acquired (see above) and previously published solubility data for vivianite in order to retrieve a single K_sp_ for vivianite as well as ionic strength-independent formation constants pertaining to the formation of Fe-phosphate complexes. Because our model accounts for Ca^2+^ and Mg^2+^ interactions with phosphate species, and also closely represents measured Fe^2+^ and phosphate anion activities as a function of pH, this approach also takes into account the differences in media composition in our synthetic seawater solutions (i.e., no calcium, sulfate or dissolved inorganic carbon) compared to that of standard seawater, and also between various studies where media composition differs. Our experimental pH measurements were calibrated against NIST (NBS) buffer standards, and so experimental measurements are reported on the NBS scale. The differences between pH values assigned on the NBS and free hydrogen ion concentration scale are small^71–73^, and, according to ref. 73, within the experimental precision of our measurements. Thus, in estimating ion activity coefficients, we specify the free ion concentration, *m*H^+^ as equal to 10^-pH(NBS)^. In this way, we derive the calculated pH as −log_10_(*a*_H+_).

Previously reported vivianite solubility data were re-calculated with our model using total Fe and phosphate concentrations and other reported experimental details. Ref. 74 did not report total Fe and P in solution during solubility determinations which precludes their raw data from being included in the optimisation procedure for Fe-phosphate complexing. However, ref. 74 reported the quantity −log([Fe]^3^*[H_2_PO_4_]^2^) versus pH. To facilitate comparison, these values were recovered by digital analysis of the published figure file. Then, activity coefficients of Fe^2+^ and H_2_PO_4_^-^ were calculated with our model at 0.1 M ionic strength (in an NaClO_4_ medium) and used to correct the published concentration products to activity products and solubility.

The optimisation procedure included describing experimentally acquired total aqueous Fe and P analyses in terms of vivianite solubility equilibrium and including as unknowns, association constants for four Fe-phosphate complexes. The resulting non-linear relations were optimised using a non-linear least squares solver in Matlab until convergence was obtained; the procedure was undertaken by constraining initial guesses for association constants to within reasonable values given pH dependence of various phosphate species.

When the optimisation procedure was completed, the resulting vivianite solubility may be represented by combining the vivianite K_sp_ reaction with the second (K_2_) and third (K_3_) dissociation constants of phosphoric acid, yielding the equation:

1 $${{{{{{\rm{Fe}}}}}}}_{3}{({{{{{{\rm{PO}}}}}}}_{4})}_{2}\bullet {8{{{{{\rm{H}}}}}}}_{2}{{{{{\rm{O}}}}}}({{{{{\rm{vivianite}}}}}})+{4{{{{{\rm{H}}}}}}}^{+}={3{{{{{\rm{Fe}}}}}}}^{2+}+{2{{{{{\rm{H}}}}}}}_{2}{{{{{{\rm{PO}}}}}}_{4}^{-}}+{8{{{{{\rm{H}}}}}}}_{2}{{{{{\rm{O}}}}}}$$

Corresponding equilibrium relationships yield:

2 $${{{\log }}\,{{{{{\rm{K}}}}}}}_{{{{{{\rm{sp}}}}}}}\,{-\,2\,{{\log }}\,{{{{{\rm{K}}}}}}}_{2}\,{-\,2\,{{\log }}\,{{{{{\rm{K}}}}}}}_{3}+4{{{{{\rm{pH}}}}}}={{\log }}\,({a}_{{{{{{\rm{Fe}}}}}}}^{3}*{a}_{{{{{{\rm{H}}}}}}2{{{{{\rm{PO}}}}}}4}^{2})$$

Rearranging into the equation for a straight line indicates that plotting our data for −log(*a*_Fe_^3^∗*a*_H2PO4_^2^) versus pH yields a straight line with a slope of 4 (dictated by vivianite stoichiometry). Determining the y-intercept and employing dissociation constants for phosphoric acid yields a vivianite solubility of pK_sp_ = 32.1140 (with 95% confidence intervals at 31.862 and 32.367; note that this is expressed in terms of Fe^2+^, PO_4_^3−^ and H_2_O). Re-casting this in terms of the solubility reaction, including HPO_4_^2−^ (K_S0_ in ref. 20), yields pK_0_ = −7.4242.

The optimised chemical equilibrium model was also used to recalculate the saturation state of vivianite in natural pore waters from published analyses (Refs. 75, 76; Supplementary Figs. 1–3). Where datasets did not report pH, it was calculated from equilibrium carbonate chemistry using dissolved inorganic carbon (DIC) analyses and total alkalinity (TA) analyses. Where DIC was not reported, we assumed an identical proportion of TA taken up by DIC, given the strong correlation between TA and DIC in comparable cores (e.g., ref. 75). It is worth noting that ref. 75 reported substitution of Mn in the vivianite structure up to a few mol%. We are not equipped with the data to evaluate the effects (if any) of Mn incorporation into vivianite, but we note here that our solubility experiments did not employ Mn in solution, yet our results generate estimated solubilities exactly in line with natural systems where at least a few mol% Mn has been incorporated (Supplementary Figs. 1–3). On this basis, we hypothesise that the effect of Mn substitution on vivianite solubility is likely negligible within the confines of our technique.

### Quantification of solubility controls on marine phosphate concentrations

To use the model to assess solubility controls on total marine phosphate concentrations, we employed seven different estimates of the major element composition (i.e., Na, K, Ca, Mg, Cl, SO_4_) of prebiotic seawater, encompassing end-member scenarios for the relative concentration of Ca and Mg, an important variable controlling the solubility of phosphate minerals in seawater. These estimates include (1) modern seawater composition at 35 permil salinity, (2) modern seawater composition at 70 permil salinity (equating to a doubling of cation and ion concentrations), (3 and 4) fluid compositions derived from water–rock interaction with komatiite at two water:rock ratios (simulating the extent of hydrothermal circulation through the ancient seafloor)^29^, (5 and 6) fluid compositions derived from water–rock interaction with basalt at two water:rock ratios (simulating the extent of hydrothermal circulation through the ancient seafloor)^29^ and (7) a fluid composition derived from elevated hydrothermal water flux and modern proportions of Mg removal at near- and off-axis vent fluids^28^. All solubility calculations specified saturation with respect to amorphous silica, and *p*CO_2_ = 0.1 bar^28^. Fe(II) concentrations under these conditions and across this pH range equate to the lowest concentrations maintained by relevant minerals. Under these conditions, at high pH (above ~7.2–7.7) greenalite limits Fe(II) solubility. Because greenalite solubility increases rapidly with decreasing pH, at pH levels below this value in some cases (for modern seawater compositions and for basalt-derived compositions, vivianite serves to control Fe(II) solubility at intermediate pH values because it is less soluble than thresholds and solubility estimates of Fe(II)-carbonate in ancient seawater25. At low pH values, all Fe(II) minerals become highly soluble and we choose to place an upper limit on Fe(II) concentrations under these conditions of 3 mmol/kg which corresponds to the lowest concentrations of fluids derived from interaction with basalt/komatiite on cooling to 25^ o^C^29^. In all cases, across this pH range, the solubility of OCP controls phosphate concentrations.

### Reaction path models of evaporation

Because the thermodynamic model described above is based on the compilation of ref. 61, we also adopt equilibrium constants for mineral solubilities reported in that study, which includes carbonate, sulfate, chloride, oxide and hydroxide minerals. In addition, we included the additional mineral species to obtain a more comprehensive representation of mineral-water interactions influencing seawater media. To maintain internal consistency, we included greenalite, using an equilibrium constant calculated from raw solubility data reported in ref. 77. Representation of FeCO_3_ precipitation was included by using the apparent log K corresponding to amorphous FeCO_3_ precipitation from carbonated seawater media as reported in ref. 25. In order to comprehensively reflect phosphate mineral solubility in seawater, we re-calculated equilibrium constants for the Mg-phosphate minerals bobbierite and newberyite (from ref. 78) and compared the solubility data for seawater solutions to that reported by ref. 79, and re-calculated equilibrium constants for octacalcium phosphate (OCP) using data reported in ref. 80.

Reaction path models of evaporation were implemented in Geochemists Workbench v15^81^ using the thermodynamic model described above. Calculations for prebiotic seawater adopted modern concentrations for Na, K, Ca and Mg; SO_4_ was not included in the calculations. Initial [SiO_2_(*aq*)] corresponded to amorphous silica saturation and total [Fe] and [PO_4_] were both set initially to 200 μmol/kg. Solutions maintained equilibrium with 0.1 bar CO_2_^23,28^ and evaporation calculations were performed at varying ratios of [ALK]/[Ca+Mg]. Backreaction with precipitated minerals was not considered in these calculations, and the following minerals were suppressed from the calculations: calcite (allowing aragonite to precipitate instead, given Mg/Ca ratios), dolomite, hydroxyapatite (instead allowing OCP to control Ca-phosphate solubility), magnesite (allowing hydrated Mg-CO_3_ phases such as nesquehonite to precipitate instead), and siderite (allowing Fe–CO_3_ precipitation to occur via AFC as discussed above). The reaction path simulations for early martian aquatic environments attempt to capture geochemical conditions recorded by sedimentary deposits analysed by landed and orbiting missions. Reaction path models examining the evaporation of waters derived from basaltic substrates employed an initial solution composition reported in ref. 82 where total ion concentrations (in μmol/kg) for Mg, Fe, Ca, Na and K are: 497, 356, 165, 25 and 10, respectively. ALK/[Ca + Mg] was modulated by varying SO4 and Cl concentrations but maintaining equal molar concentrations. All solutions maintained equilibrium with 1 bar CO_2_. We neglect appreciable concentrations of Al in our model as well as reduced sulfur species because in-situ and orbital data acquired from ancient martian rocks provide little evidence for (1) significant Al mobility, or (2) authigenic precipitation of Fe-sulfide minerals at surface conditions^83^.

### Reporting summary

Further information on research design is available in the Nature Research Reporting Summary linked to this article.

## Supplementary information

Supplementary Information

Reporting Summary

## Data Availability

The authors declare that the data supporting the findings of this study are available within the paper and its Supplementary information files.

## Code availability

Optimisation of solubility/thermodynamic data was performed using custom scripts referring to algorithms included with licensed Matlab (v2020b) software; these are available on request from the corresponding author.
