# Supplementary material for: Marine phosphate availability and the chemical origins of life on Earth
Source: Nat Commun. 2022 Sep 2;13:5162. doi: 10.1038/s41467-022-32815-x (PMC9440033; doi:10.1038/s41467-022-32815-x)
Supplement: Supplementary file 1 — Supplementary Information [file 41467_2022_32815_MOESM1_ESM.pdf]

Marine phosphate availability and the chemical origins of life on Earth: Supplementary Information

Supplementary figures 1-9
Supplementary tables 1 & 2

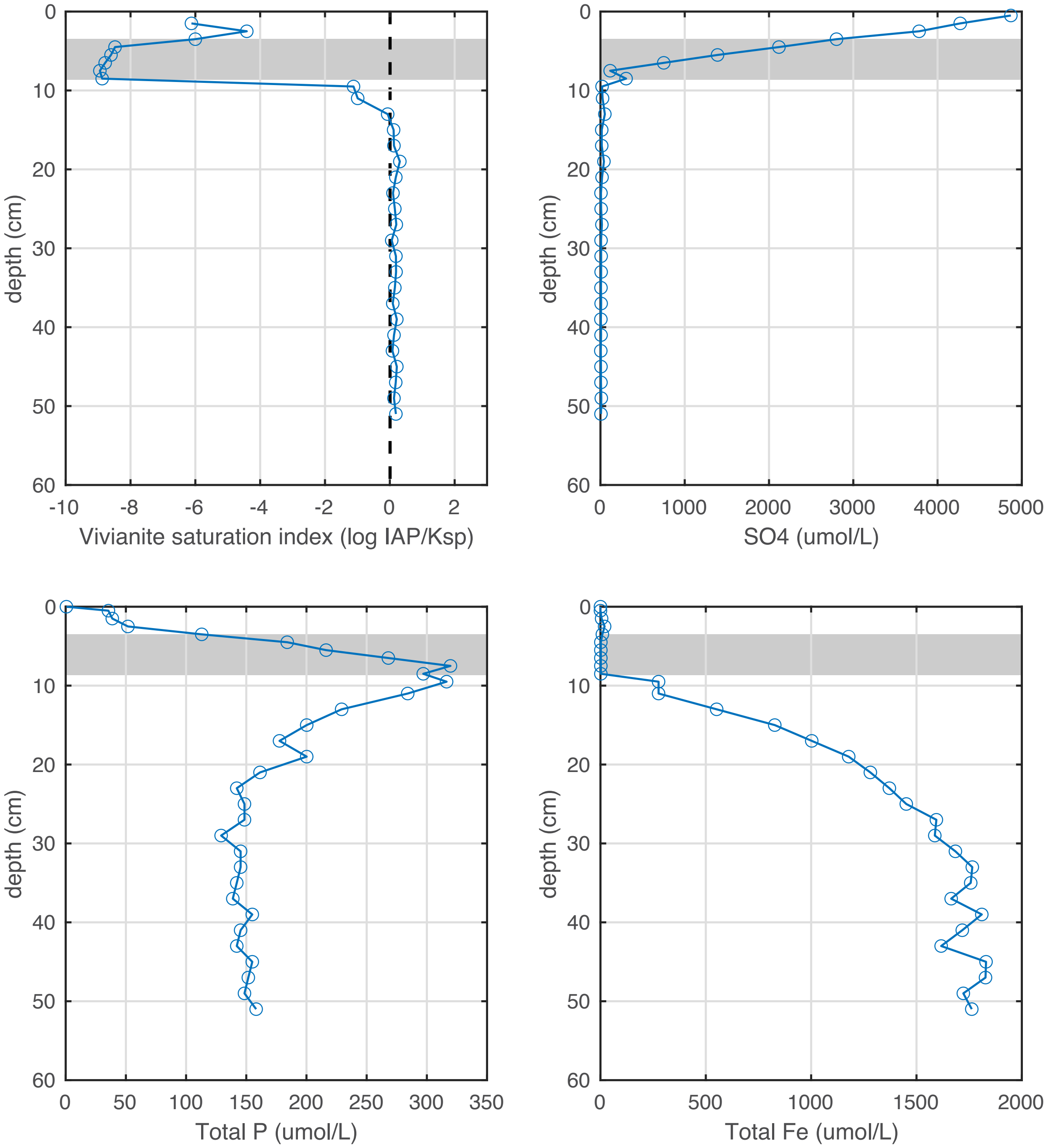

Supplementary figure 1. Recalculation of vivianite saturation index for sediment pore waters extracted from cores sampled from site US5B in the Bothnian Sea (2012 data)[75]. Ref 75 identified and quantified vivianite within the cores using sequential extraction, XRF, SEM-EDS, XRD and synchrotron-based XANES. Grey bar indicates sulfate-methane transition zone (SMTZ), below which ref 75 report the presence of vivianite. Model-calculated vivianite saturation index (upper left panel), using the model developed herein, shows that despite variable [Fe] and phosphate, pore water chemistry below the SMTZ is poised slightly above vivianite solubility equilibrium, consistent with vivianite preservation in these sediments.

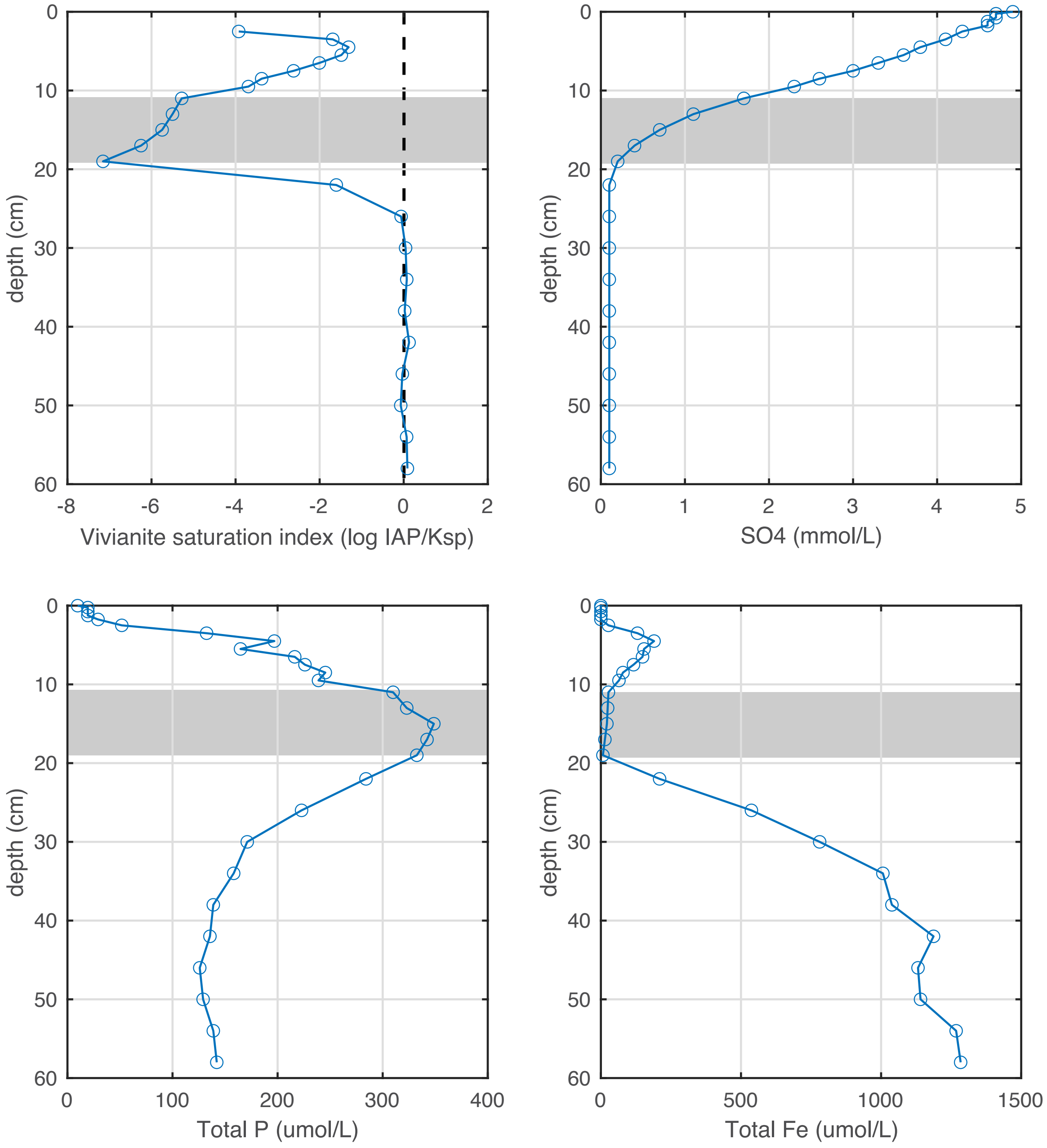

Supplementary figure 2. Recalculation of vivianite saturation index for sediment pore waters extracted from cores sampled from site US5B in the Bothnian Sea (2013 data)[75]. Ref 75 identified and quantified vivianite within the cores using sequential extraction, XRF, SEM-EDS, XRD and synchrotron-based XANES. Grey bar indicates sulfate-methane transition zone (SMTZ), below which ref 75 report the presence of vivianite. Model-calculated vivianite saturation index (upper left panel), using the model developed herein, shows that despite variable [Fe] and phosphate, pore water chemistry below the SMTZ is poised slightly above vivianite solubility equilibrium, consistent with vivianite preservation in these sediments.

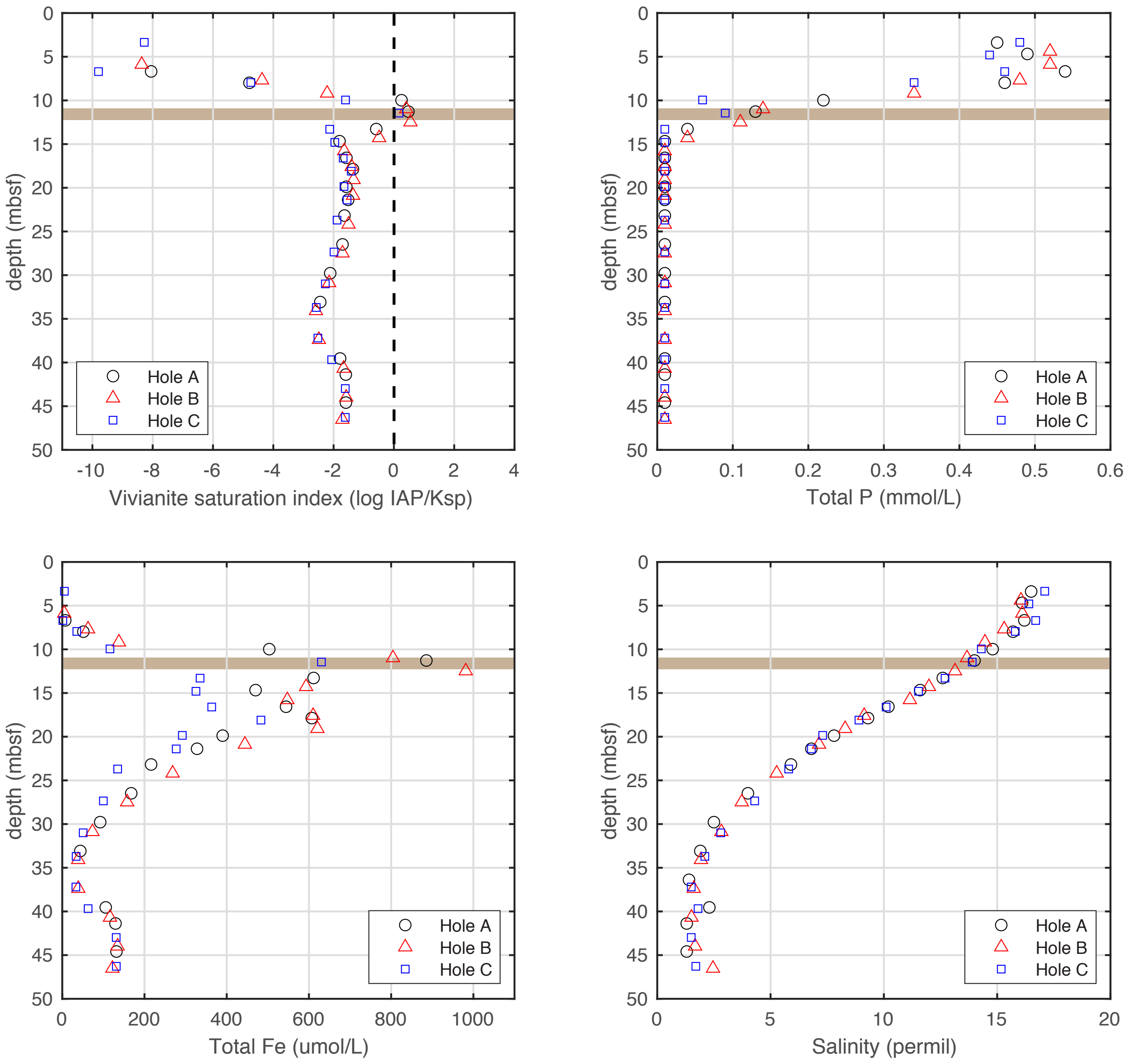

Supplementary figure 3. Recalculation of vivianite saturation index for sediment pore waters extracted from cores sampled from site M0065 in the Bornholm Basin[85]. Ref 85 identified and quantified vivianite within the cores using sequential extraction, SEM-EDS, and synchrotron-based XANES. Brown bar indicates a peak in sediment-bound P, which serves as a proxy for vivianite abundance. Model-calculated vivianite saturation index (upper left panel), using the model developed herein, shows that despite variable [Fe], phosphate and salinity, pore water chemistry is poised slightly above vivianite solubility equilibrium only in the location the mineral is observed.

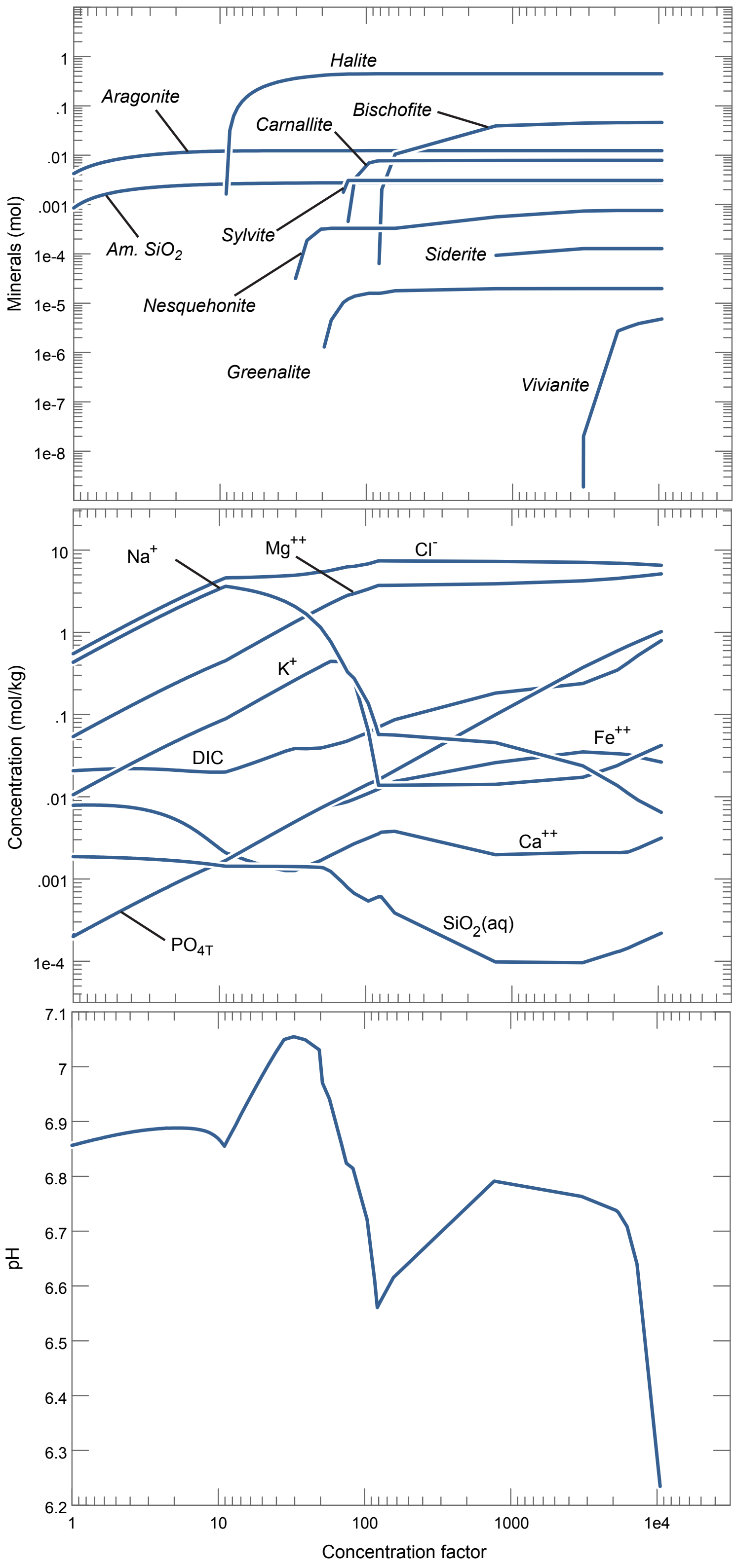

Supplementary figure 4. Reaction path model of seawater evaporation at 25°C and pCO_2 = 0.1 bar. This calculation excludes back reaction of minerals with the aqueous fluid. Suppressed phases: dolomite, calcite, magnesite. Initial cation composition set to modern values and initial ALK/[Ca + Mg] = 0.96. Details of the thermodynamic model, including activity coefficient estimation and mineral solubility products are described in the Methods section. Aqueous concentrations refer to total cation and anions; concentrations of individual aqueous species included in the model are omitted for clarity. This calculation is also plotted on Figure 3 of the manuscript.

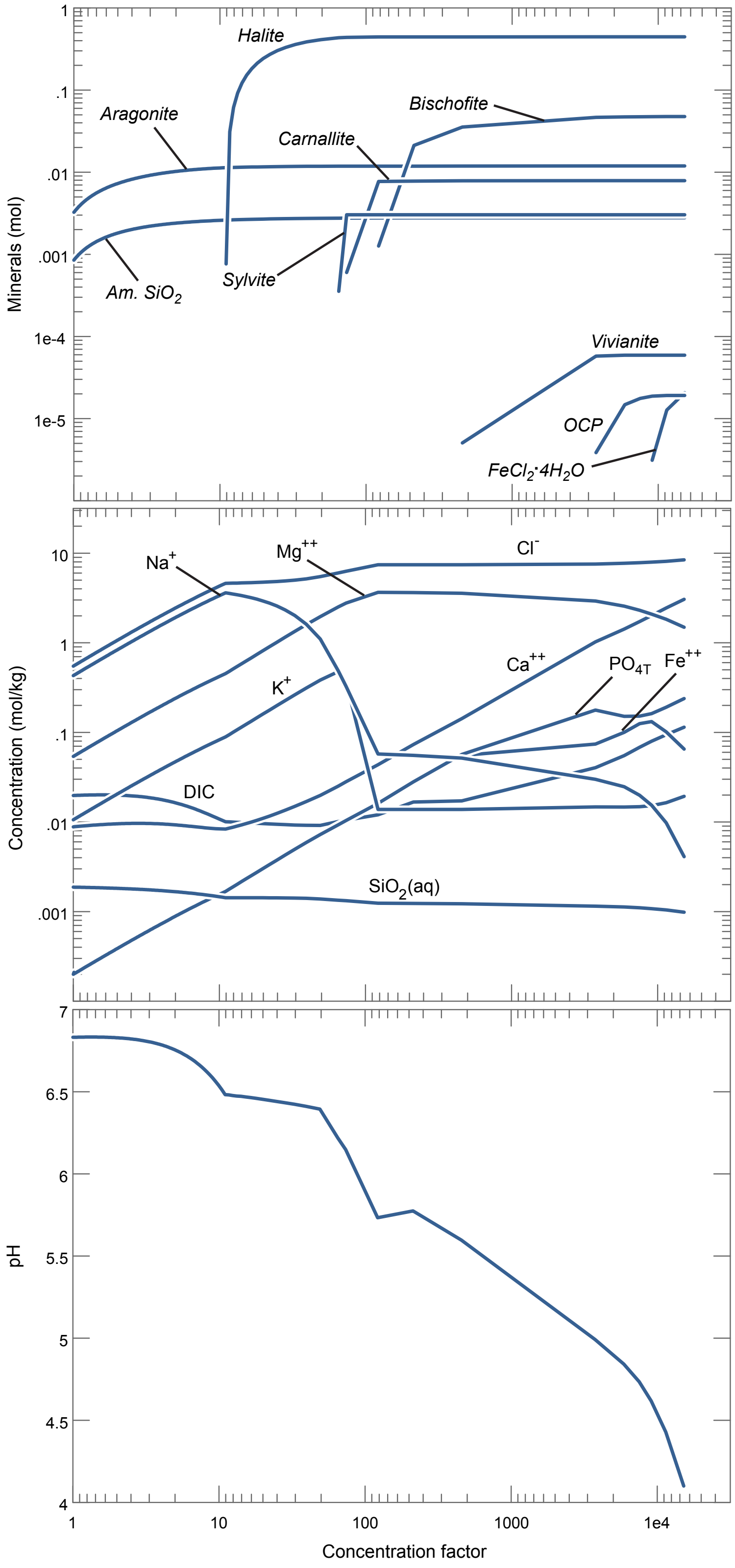

Supplementary figure 5. Reaction path model of seawater evaporation at 25°C and pCO_2 = 0.1 bar. This calculation excludes back reaction of minerals with the aqueous fluid. Suppressed phases: dolomite, calcite, magnesite. Initial cation composition set to modern values and initial ALK/[Ca + Mg] = 0.74. Details of the thermodynamic model, including activity coefficient estimation and mineral solubility products are described in the Methods section. Aqueous concentrations refer to total cation and anions; concentrations of individual aqueous species included in the model are omitted for clarity. This calculation is also plotted on Figure 3 of the manuscript.

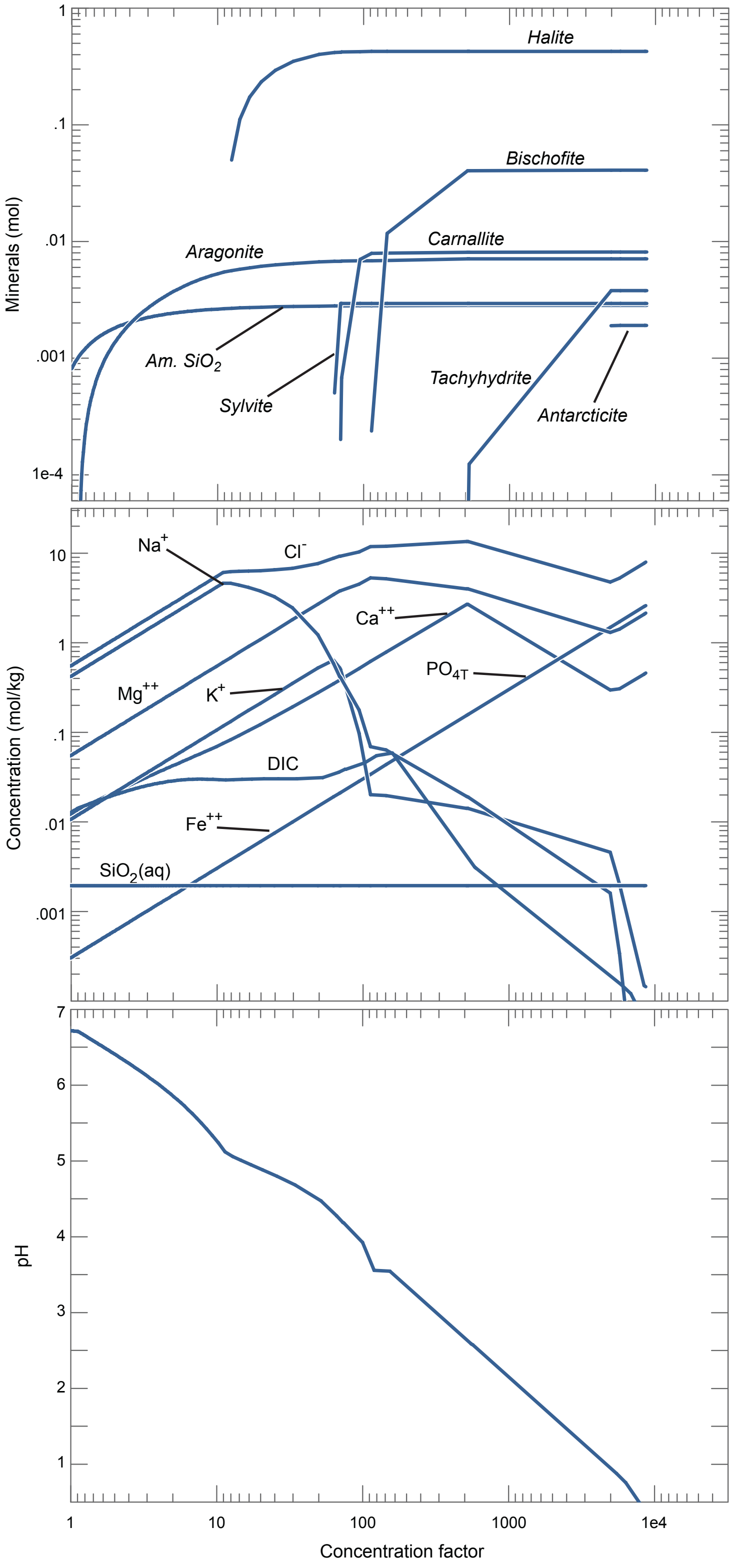

Supplementary figure 6. Reaction path model of seawater evaporation at 25°C and pCO_2 = 0.1 bar. This calculation excludes back reaction of minerals with the aqueous fluid. Suppressed phases: dolomite, calcite, magnesite. Initial cation composition set to modern values and initial ALK/[Ca + Mg] = 0.11. Details of the thermodynamic model, including activity coefficient estimation and mineral solubility products are described in the Methods section. Aqueous concentrations refer to total cation and anions; concentrations of individual aqueous species included in the model are omitted for clarity. This calculation is also plotted on Figure 3 of the manuscript.

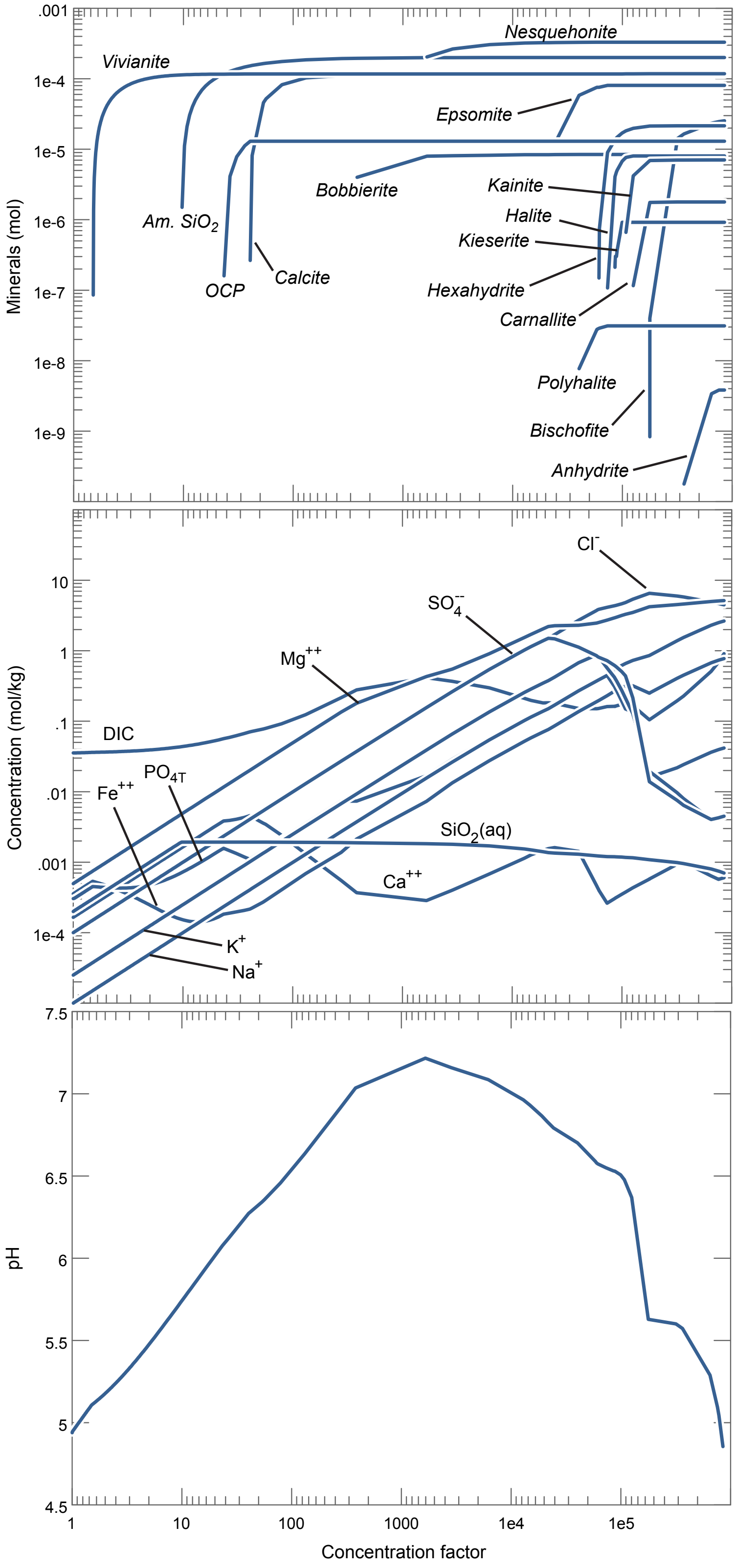

Supplementary figure 7. Reaction path model of evaporation of water derived from interaction with martian basalt[80] at 25°C and pCO_2 = 1 bar. This calculation excludes back reaction of minerals with the aqueous fluid. Suppressed phases: dolomite, calcite, magnesite. Initial cation composition set to modern values and initial ALK/[Ca + Mg] = 2.20. Details of the thermodynamic model, including activity coefficient estimation and mineral solubility products are described in the Methods section. Aqueous concentrations refer to total cation and anions; concentrations of individual aqueous species included in the model are omitted for clarity. This calculation is also plotted on Figure 3 of the manuscript.

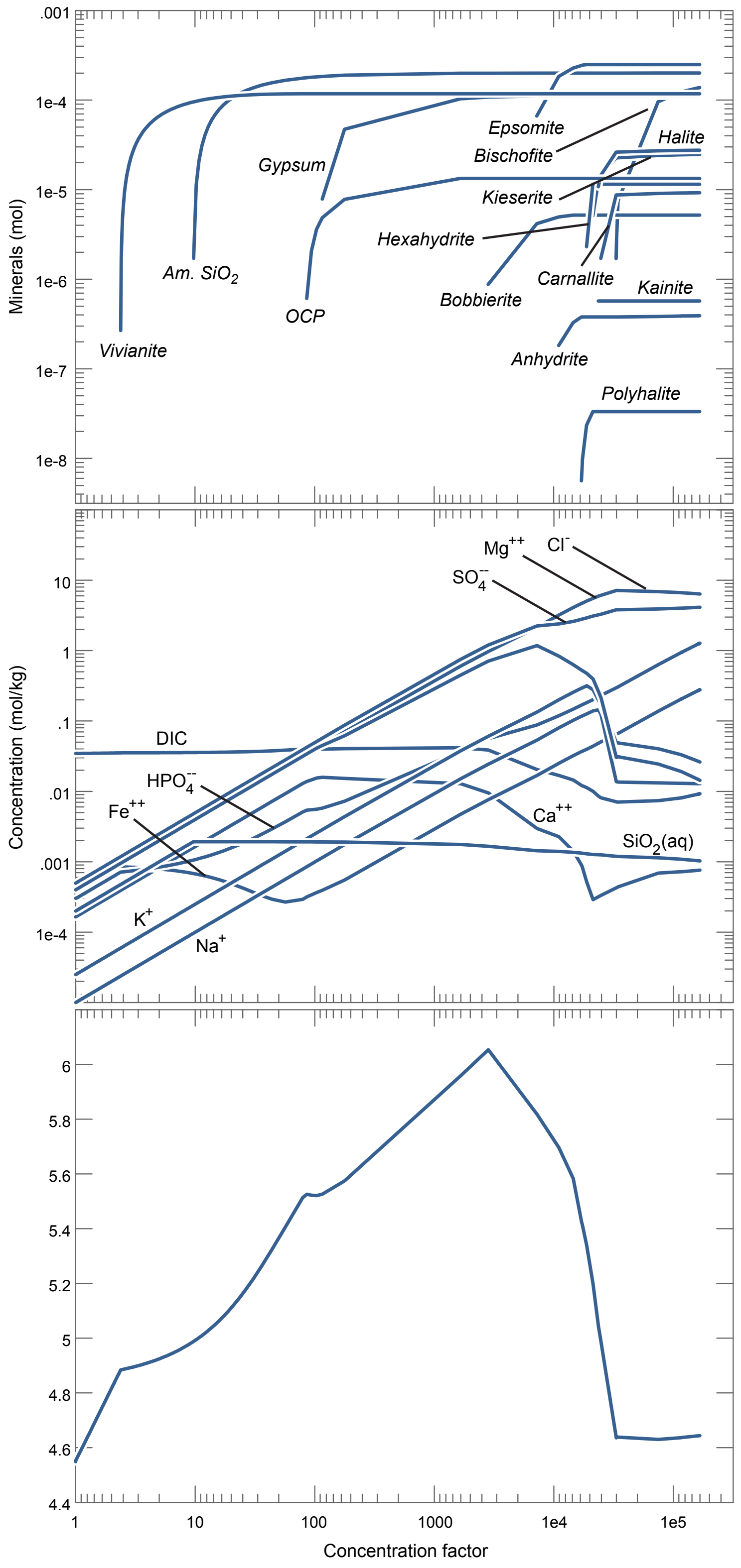

Supplementary figure 8. Reaction path model of evaporation of water derived from interaction with martian basalt[80] at 25°C and pCO_2 = 1 bar. This calculation excludes back reaction of minerals with the aqueous fluid. Suppressed phases: dolomite, calcite, magnesite. Initial cation composition set to modern values and initial ALK/[Ca + Mg] = 1.33. Details of the thermodynamic model, including activity coefficient estimation and mineral solubility products are described in the Methods section. Aqueous concentrations refer to total cation and anions; concentrations of individual aqueous species included in the model are omitted for clarity. This calculation is also plotted on Figure 3 of the manuscript.

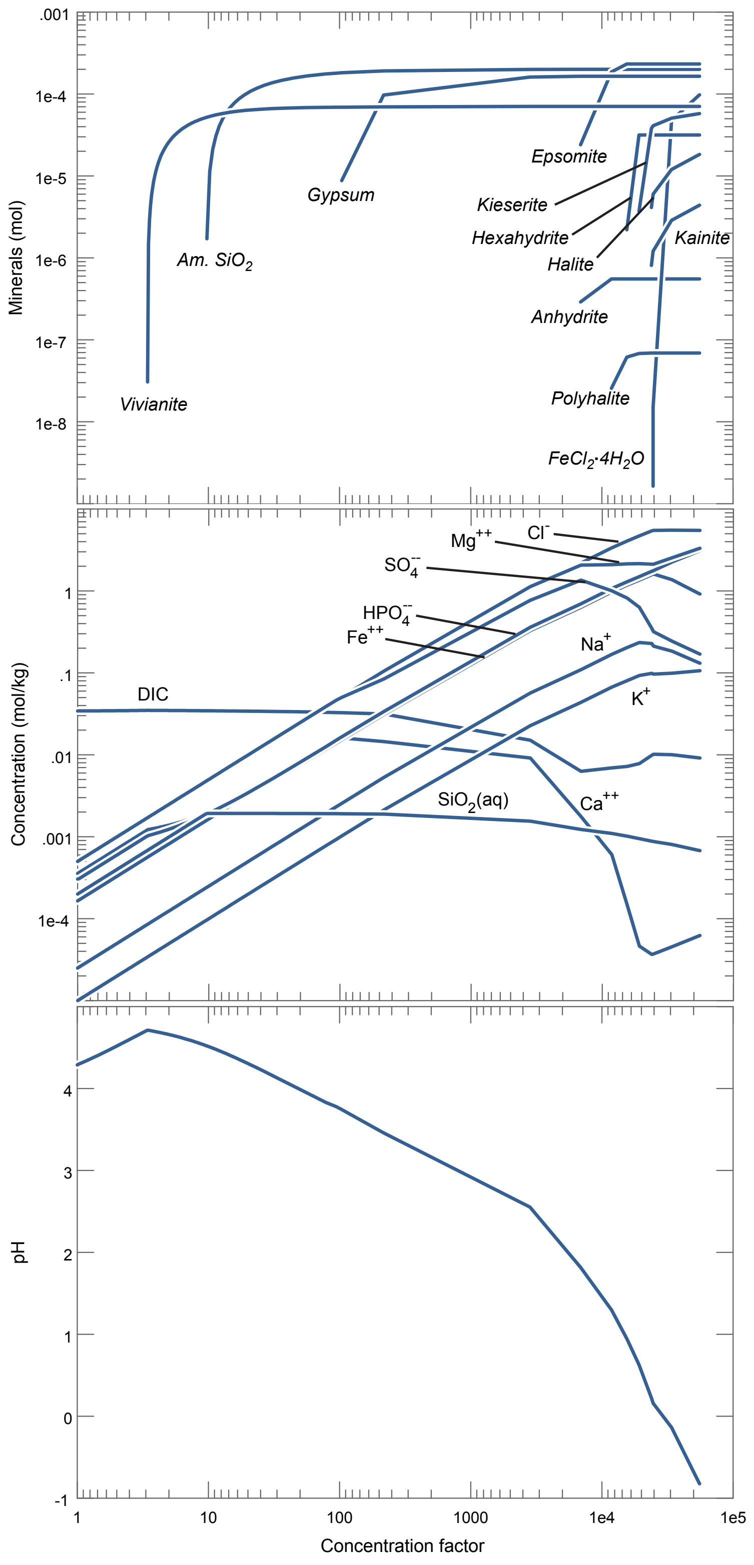

Supplementary figure 9. Reaction path model of evaporation of water derived from interaction with martian basalt[80] at 25°C and pCO_2 = 1 bar. This calculation excludes back reaction of minerals with the aqueous fluid. Suppressed phases: dolomite, calcite, magnesite. Initial cation composition set to modern values and initial ALK/[Ca + Mg] = 0.12. Details of the thermodynamic model, including activity coefficient estimation and mineral solubility products are described in the Methods section. Aqueous concentrations refer to total cation and anions; concentrations of individual aqueous species included in the model are omitted for clarity. This calculation is also plotted on Figure 3 of the manuscript.

Supplementary Table 1. Experimental measurements of vivianite solubility (25°C) and selected model-calculated ionic activities

Total P (mmolal)	Error (abs.)	Total Fe (mmolal)	Error (abs.)	pH (NBS scale)	Error (abs.)	pH (calculated)	log aFe2+	log aPO43-	log aHPO4	aH2O	Ionic Strength	log K_{sp} Vivianite	Source	Note
0.260	0.020	0.289	0.010	8.00	0.05	8.11	-4.80	-10.61	-6.38	0.98	0.69	-35.7	This study	1
0.270	0.020	0.289	0.010	8.00	0.05	8.11	-4.83	-10.58	-6.35	0.98	0.69	-35.7	This study	1
0.270	0.020	0.290	0.010	8.00	0.05	8.11	-4.83	-10.58	-6.35	0.98	0.69	-35.7	This study	1
0.260	0.020	0.289	0.010	8.00	0.05	8.11	-4.80	-10.61	-6.38	0.98	0.69	-35.7	This study	1
0.250	0.020	0.230	0.010	7.50	0.05	7.62	-4.90	-10.73	-6.00	0.98	0.69	-36.2	This study	1
0.240	0.020	0.235	0.010	7.51	0.05	7.63	-4.85	-10.77	-6.05	0.98	0.69	-36.2	This study	1
0.033	0.020	0.465	0.010	8.36	0.05	8.47	-4.09	-12.02	-8.15	0.98	0.69	-36.4	This study	1
0.052	0.020	0.459	0.010	8.34	0.05	8.45	-4.11	-11.80	-7.91	0.98	0.69	-36.0	This study	1
0.057	0.020	0.457	0.010	8.33	0.05	8.44	-4.12	-11.76	-7.85	0.98	0.69	-36.0	This study	1
0.430	0.020	1.170	0.010	6.73	0.05	6.78	-3.81	-12.03	-6.47	0.96	1.54	-35.6	This study	1
0.450	0.020	1.170	0.010	6.69	0.05	6.74	-3.81	-12.04	-6.43	0.96	1.54	-35.7	This study	1
0.430	0.020	1.150	0.010	6.68	0.05	6.73	-3.82	-12.05	-6.44	0.96	1.54	-35.7	This study	1
0.410	0.020	1.150	0.010	6.66	0.05	6.71	-3.81	-12.08	-6.44	0.96	1.54	-35.7	This study	1
1.891	0.019	3.001	0.030	4.72	0.05	4.72	-3.15	-12.95	-5.32	1.00	0.06	-35.4	20	2
1.639	0.016	2.762	0.028	4.71	0.05	4.71	-3.18	-13.03	-5.39	1.00	0.05	-35.6	20	2
1.587	0.016	2.677	0.027	4.71	0.05	4.71	-3.20	-13.04	-5.40	1.00	0.05	-35.7	20	2
1.582	0.016	2.641	0.026	4.70	0.05	4.70	-3.20	-13.05	-5.41	1.00	0.05	-35.7	20	2
1.587	0.016	2.645	0.026	4.71	0.05	4.71	-3.20	-13.04	-5.40	1.00	0.05	-35.7	20	2
1.512	0.015	2.508	0.025	4.71	0.05	4.70	-3.23	-13.07	-5.43	1.00	0.05	-35.8	20	2
1.324	0.013	2.488	0.025	4.70	0.05	4.70	-3.23	-13.14	-5.49	1.00	0.05	-36.0	20	2

1.201	0.012	2.203	0.022	4.70	0.05	4.70	-3.28	-13.18	-5.53	1.00	0.05	-36.2	20	2
3.180	0.048	1.560	0.031	4.42	0.01	4.42	-2.94	-13.24	-5.32	1.00	0.00	-35.3	78	--
2.300	0.035	1.130	0.023	4.51	0.01	4.51	-3.07	-13.20	-5.36	1.00	0.00	-35.6	78	--
2.150	0.032	1.060	0.021	4.57	0.01	4.57	-3.09	-13.11	-5.33	1.00	0.00	-35.5	78	--
0.881	0.013	0.436	0.009	4.96	0.01	4.96	-3.45	-12.71	-5.32	1.00	0.00	-35.8	78	--
0.771	0.012	0.384	0.008	5.04	0.01	5.04	-3.50	-12.61	-5.30	1.00	0.00	-35.7	78	--
0.542	0.008	0.269	0.005	5.15	0.01	5.15	-3.65	-12.54	-5.35	1.00	0.00	-36.0	78	--
0.454	0.007	0.226	0.005	5.24	0.01	5.24	-3.73	-12.44	-5.34	1.00	0.00	-36.1	78	--
0.382	0.006	0.191	0.004	5.32	0.01	5.32	-3.80	-12.36	-5.33	1.00	0.00	-36.1	78	--
0.324	0.005	0.162	0.003	5.40	0.01	5.40	-3.88	-12.27	-5.33	1.00	0.00	-36.2	78	--
0.302	0.005	0.151	0.003	5.49	0.01	5.49	-3.92	-12.13	-5.28	1.00	0.00	-36.0	78	--
0.255	0.004	0.129	0.003	5.69	0.01	5.69	-4.04	-11.84	-5.18	1.00	0.00	-35.8	78	--

[1]Ca- and SO_4-free synthetic seawater solution
[2]0.05 molal acetic acid / 0.05 molal Na-acetate buffer

Supplementary Table 2. Acid dissociation constants, formation constants, and equilibrium constants (25°C) for aqueous phosphate species and phosphate minerals considered in the model.

Reaction	log K	Source
$H_3PO_4^o = H^+ + H_2PO_4^-$	-2.1490	59
$H_2PO_4^- = H^+ + HPO_4^{2-}$	-7.1998	59
$HPO_4^{2-} = PO_4^{3-} + H^+$	-12.345	59
$Ca^{2+} + H_2PO_4^- = CaH_2PO_4^+$	-1.0	63
$Ca^{2+} + HPO_4^{2-} = CaHPO_4^o$	-2.66	63
$Ca^{2+} + PO_4^{3-} = CaPO_4^-$	-6.8	63
$Mg^{2+} + HPO_4^{2-} = MgHPO_4^o$	-2.8	63
$Mg^{2+} + H_2PO_4^- = MgH_2PO_4^+$	-1.13	63
$Mg^{2+} + PO_4^{3-} = MgPO_4^-$	-5.63	63
$Fe^{2+} + HPO_4^{2-} = FeHPO_4^o$	-2.0	This study
$Fe^{2+} + H_2PO_4^- = FeH_2PO_4^+$	2.2	This study
$Fe^{2+} + HPO_4^{2-} + PO_4^{3-} = Fe(HPO_4)(PO_4)^{3-}$	10.3	This study
$Fe^{2+} + PO_4^{3-} = FePO_4^-$	-11.3	This study
$Fe_3(PO_4)_2{\bullet}8H_2O$ (*vivianite*) $+2H^+ = 3Fe^{2+} + 2HPO_4^{2-} + 8H_2O$	-7.4242	This study
$Ca_4H(PO_4)_3{\bullet}2.25H_2O$ (*OCP*) $+2H^+ = 4Ca^{2+} + 3HPO_4^{2-} + 2.25H_2O$	-11.806	Recalculated from solubility data from 78
$MgHPO_4{\bullet}3H_2O$ (*newberyite*) $= Mg^{2+} + HPO_4^{2-} + 3H_2O$	-5.8198	88
$Mg_3(PO_4)_2{\bullet}8H_2O$ (*bobbierite*) $+2H^+ = 3Mg^{2+} + 2HPO_4^{2-} + 8H_2O$	-0.4996	88
