# Supplementary material for: Marine phosphate availability and the chemical origins of life on Earth
Source: Nat Commun. 2022 Sep 2;13:5162. doi: 10.1038/s41467-022-32815-x (PMC9440033; doi:10.1038/s41467-022-32815-x)
Supplement: Supplementary file 2 — Reporting Summary [file 41467_2022_32815_MOESM2_ESM.pdf]

nature portfolio

Corresponding author(s): Nicholas Tosca (njt41@cam.ac.uk)

Last updated by author(s): 9 August 2022

Reporting Summary

Nature Portfolio wishes to improve the reproducibility of the work that we publish. This form provides structure for consistency and transparency in reporting. For further information on Nature Portfolio policies, see our Editorial Policies and the Editorial Policy Checklist.

Statistics

For all statistical analyses, confirm that the following items are present in the figure legend, table legend, main text, or Methods section.

n/a	Confirmed	
☐	☒	The exact sample size (*n*) for each experimental group/condition, given as a discrete number and unit of measurement
☐	☒	A statement on whether measurements were taken from distinct samples or whether the same sample was measured repeatedly
☐	☒	The statistical test(s) used AND whether they are one- or two-sided<br>*Only common tests should be described solely by name; describe more complex techniques in the Methods section.*
☒	☐	A description of all covariates tested
☐	☒	A description of any assumptions or corrections, such as tests of normality and adjustment for multiple comparisons
☒	☐	A full description of the statistical parameters including central tendency (e.g. means) or other basic estimates (e.g. regression coefficient) AND variation (e.g. standard deviation) or associated estimates of uncertainty (e.g. confidence intervals)
☒	☐	For null hypothesis testing, the test statistic (e.g. *F*, *t*, *r*) with confidence intervals, effect sizes, degrees of freedom and *P* value noted<br>*Give P values as exact values whenever suitable.*
☒	☐	For Bayesian analysis, information on the choice of priors and Markov chain Monte Carlo settings
☒	☐	For hierarchical and complex designs, identification of the appropriate level for tests and full reporting of outcomes
☒	☐	Estimates of effect sizes (e.g. Cohen's *d*, Pearson's *r*), indicating how they were calculated

Our web collection on statistics for biologists contains articles on many of the points above.

Software and code

Policy information about availability of computer code

Data collection	Solubility data were acquired using licensed Agilent ICP Expert software; FT-IR data on solids was collected using licensed Spectrum 10 software; XRD data on solids were acquired using licensed Panalytical HighScore software. Thermodynamic calculations and reaction path models were performed using licensed Geochemists Workbench v15 software.
Data analysis	Data were analysed using open source GSAS-II software for structural refinement of powder X-ray diffraction patterns, licensed Panalytical HighScore software for solid purity analysis of XRD data, and optimisation of solubility/thermodynamic data was performed using algorithms included with licensed Matlab v2020b software.

For manuscripts utilizing custom algorithms or software that are central to the research but not yet described in published literature, software must be made available to editors and reviewers. We strongly encourage code deposition in a community repository (e.g. GitHub). See the Nature Portfolio guidelines for submitting code & software for further information.

Data

Policy information about availability of data

All manuscripts must include a data availability statement. This statement should provide the following information, where applicable:

- Accession codes, unique identifiers, or web links for publicly available datasets
- A description of any restrictions on data availability
- For clinical datasets or third party data, please ensure that the statement adheres to our policy

The authors declare that the data supporting the findings of this study are available within the paper and its Supplementary information files.

Human research participants

Policy information about studies involving human research participants and Sex and Gender in Research.

Reporting on sex and gender	N/A
Population characteristics	N/A
Recruitment	N/A
Ethics oversight	N/A

Note that full information on the approval of the study protocol must also be provided in the manuscript.

Field-specific reporting

Please select the one below that is the best fit for your research. If you are not sure, read the appropriate sections before making your selection.

☐ Life sciences ☐ Behavioural & social sciences ☒ Ecological, evolutionary & environmental sciences

For a reference copy of the document with all sections, see nature.com/documents/nr-reporting-summary-flat.pdf

Ecological, evolutionary & environmental sciences study design

All studies must disclose on these points even when the disclosure is negative.

Study description	We determined the solubility of Fe(II)-phosphate in synthetic seawater as a function of pH and ionic strength, integrated these observations into a thermodynamic model that predicts phosphate concentrations across a range of aquatic conditions, and validated these predictions against modern anoxic sediment pore waters where Fe(II)-phosphates are present.
Research sample	Newly collected solubility data of Fe(II)-phosphates determined under anoxic conditions; modeling data obtained using licensed geochemical reaction path modeling software.
Sampling strategy	Sampling strategy was designed to cover a range of the principal factors governing Fe(II)-phosphate solubility in marine systems, including pH, metal concentration, and ionic strength.
Data collection	Data were collected by conducting solubility experiments, withdrawing filtered solution samples, determining solid phase composition and purity.
Timing and spatial scale	Data were collected over the course of solubility experiments which generally ran until solution data indicated no observable change in solution compositions, which typically took a few days to a few weeks. Solution samples were collected periodically (typically every 12 to 24 hours). Solid samples were collected at termination of the experiments.
Data exclusions	N/A
Reproducibility	Multiple solubility determinations were made within a narrow range of parameters (keeping them constant) during successive repetitions of experiments.
Randomization	N/A
Blinding	N/A

Did the study involve field work? ☐ Yes ☒ No

Reporting for specific materials, systems and methods

We require information from authors about some types of materials, experimental systems and methods used in many studies. Here, indicate whether each material, system or method listed is relevant to your study. If you are not sure if a list item applies to your research, read the appropriate section before selecting a response.

Materials & experimental systems

n/a	Involved in the study
☒	☐ Antibodies
☒	☐ Eukaryotic cell lines
☒	☐ Palaeontology and archaeology
☒	☐ Animals and other organisms
☒	☐ Clinical data
☒	☐ Dual use research of concern

Methods

n/a	Involved in the study
☒	☐ ChIP-seq
☒	☐ Flow cytometry
☒	☐ MRI-based neuroimaging
